# Wnt signaling regulates trans-differentiation of stem cell like type 2 alveolar epithelial cells to type 1 epithelial cells

**DOI:** 10.1186/s12931-019-1176-x

**Published:** 2019-09-06

**Authors:** Elhusseiny Mohamed Mahmud Abdelwahab, Judit Rapp, Diana Feller, Veronika Csongei, Szilard Pal, Domokos Bartis, David R. Thickett, Judit Erzsebet Pongracz

**Affiliations:** 1 Department of Pharmaceutical Biotechnology, School of Pharmacy, University of Pecs, 2 Rokus Str, Pecs, H-7624 Hungary; 2 Szentagothai Research Centre, University of Pecs, 20 Ifjusag Str, Pecs, H-7624 Hungary; 3 Department of Pharmaceutical Technology, School of Pharmacy, University of Pecs, 2 Rokus Str, Pecs, H-7624 Hungary; 4 Respiratory Research Group, Institute of Inflammation and Aging, University of Birmingham, Birmingham, B15 2TT UK

**Keywords:** Wnt signaling, Alveolar epithelial cell, Transdifferentiation

## Abstract

**Background:**

Type 2 alveolar epithelial cells (AT2s) behave as stem cells and show clonal proliferation upon alveolar injury followed by trans-differentiation (TD) into Type 1 alveolar epithelial cells (AT1s). In the present study we identified signaling pathways involved in the physiological AT2-to-AT1 TD process.

**Methods:**

AT2 cells can be isolated from human lungs and cultured in vitro where they undergo TD into AT1s. In the present study we identified signaling pathways involved in the physiological AT2-to-AT1 TD process using Affymetrix microarray, qRT-PCR, fluorescence microscopy, and an in vitro lung aggregate culture.

**Results:**

Affymetrix microarray revealed Wnt signaling to play a crucial role in the TD process. Wnt7a was identified as a ligand regulating the AT1 marker, Aquaporin 5 (AQP5). Artificial Neural Network (ANN) analysis of the Affymetrix data exposed ITGAV: Integrin alpha V (ITGAV), thrombospondin 1 (THBS1) and epithelial membrane protein 2 (EMP2) as Wnt signaling targets.

**Conclusions:**

Wnt signaling targets that can serve as potential alveolar epithelial repair targets in future therapies of the gas exchange surface after injury. As ITGAV is significantly increases during TD and is regulated by Wnt signaling, ITGAV might be a potential target to speed up the alveolar healing process.

## Background

Understanding the molecular regulation of alveolar regeneration is of high clinical importance. Mechanical injury of the alveoli induced by ventilation [1] or loss of gas exchange surface due to accumulation of environmental damage during aging [2] could both be treated if the process is understood and the appropriate molecular targets are identified for drug development [3].

The enormous alveolar surface of the lung has a significant and physiological regeneration capacity [4, 5]. Type 2 alveolar epithelial cells (AT2s) have been suspected to act as progenitor cells in the alveoli and recent genetic fate-tracking experiments in transgenic mice provided evidence that AT2s are indeed function as stem cells and show clonal proliferation in response to injury [6]. About 95% of the alveolar surface area is covered by flat and thin Type 1 alveolar epithelial cells (AT1) that die by apoptosis upon injury leaving a denuded alveolar basement membrane behind. The cuboid AT2s are more resistant to injury, they proliferate, migrate and spread over the basement membrane then transdifferentiate into AT1 cells [7]. The above process can happen in vitro also that was established mainly in animal studies [8, 9]. Recent organ regeneration studies suggest that reactivation of developmental mechanisms occur during injury repair [10] involving BMP, FGF, Notch and Wnt [11] signaling pathways.

Wnt/beta-catenin signaling is an evolutionarily conserved, versatile and highly complex pathway. Activation of this pathway leads to the accumulation of beta-catenin in the cytosol and translocation to the nucleus where it promotes transcription of various genes. Regulation of beta-catenin protein stability is dependent on its phosphorylation at various phosphorylation sites that either promotes protein degradation (Ser33, Ser37 and Thr41) or its stabilization and nuclear localization (Ser675) [12]. The Wnt family of secreted glycoproteins are known regulators of cell proliferation, differentiation, polarity, adhesion and migration during lung development [13]. While the Wnt/beta-catenin signaling is necessary for alveolar morphogenesis it is not essential for the development of proximal airways [14]. Several additional Wnt ligands, such as Wnt5a [15], Wnt7b [16] and most Frizzled (Fzd) receptors [17] are also central to the regulation of lung development. During aging deregulated Wnt ligand composition can alter alveolar epithelial differentiation [18] and can give rise to modified molecular microenvironments that promote emphysema and other diseases [19]. Although it is recognized that Wnt signaling must have a critical role in pulmonary regeneration, its precise involvement in the trans-differentiation (TD) processes remain obscure. Particularly so, as most of the studies were performed in cell line, mouse and rat models using immunostaining [20, 21] which did now allow a preconception free approach to understand the role of Wnt signaling of AT2-to-AT1 TD in the human lung. To investigate the process, cellular transformation of primary human AT2 cells was studied in vitro and data was compared to gene expression of AT1 and AT2 cells freshly isolated from primary human lung tissues. The effects of the identified Wnt ligands were tested in three-dimensional (3D) human lung aggregate cultures [22] to confirm their roles in the TD process and in the regulation of their downstream targets recognized by artificial neural network (ANN) analysis.

## Materials and methods

### Cell isolation from lung tissue

Human primary lung epithelial cells were isolated from tissue samples from lobectomy patients (*n* = 26) with normal lung function. All patients gave written informed consent for the use of their tissues and clinical data for research purposes (ethics 07/MRE08/42). Patient characteristics are summarized in Additional file 1: Table S1. Cells were isolated and cultured as described previously [23]. Briefly, lung tissue was rinsed with saline, then digested using warm trypsin, followed by mincing in the presence of DNase (Sigma-Aldrich, St. Louis, Missouri, USA) and foetal calf serum (FCS) (Lonza, Basel, Switzerland). The cell suspension was passed through a cell strainer (mesh size 40 μm) and the freshly isolated cells were either processed for Fluorescent activated cell sorting (FACS) or cultured for longer term [24].

### In vitro TD of primary pulmonary epithelial cells

Pulmonary epithelial cells were seeded into collagen coated plates and cultured in DCCM-1 medium (Biological Industries Ltd. Kibbutz Beit-Haemek, Israel) containing 10% FCS for 3–6 days. On day 3 and day 6, cells were lysed and total RNA was isolated and processed for microarray analysis or real-time qPCR.

### Sorting of freshly isolated lung epithelial cells

Freshly isolated lung cells (*n* = 12) were washed with PBS containing 0.1% BSA (Sigma-Aldrich, St. Louis, Missouri, USA) and 0.1% Na-Azide (Sigma-Aldrich, St. Louis, Missouri, USA), then antibodies were added for 30 min. EpCAM-FITC, CD208-PE and Podoplanin-APC conjugated antibodies were used to differentiate between AT1-like (EpCAM+ Podoplanin+ CD208- population) [25] and AT2-like (EpCAM+ Podoplanin- CD208+ population) [26] epithelial cells. Cells were sorted with a Beckman-Coulter MoFlo XDP high-speed cell sorter (Additional file 1: Figure S1). Average yield of AT1- and AT2-like cells were 4.43 × 10^4^ and 1.39 × 10^5^, respectively. Cells were then lysed, RNA was isolated using RNeasy kit (Qiagen, Hilden, Germany) and cDNA was generated from 200 ng total RNA using a WT Expression Kit (Ambion, Thermo Fisher Scientific, Waltham, USA).

### Microarray analysis

cDNA of in vitro cultured cells of *n* = 3 patients (days 3 and 6 of culturing) was fragmented and fluorescently labeled using the GeneChip WT terminal Labeling Kit (Affymetrix, Santa Clara, USA). cDNA was hybridized to Human Gene 1.0 ST arrays (Affymetrix, Santa Clara, USA). Probe cell intensity data (CEL) from the microarrays were analysed using the Expression Console software with the default settings of the RMA-sketch workflow. Differentially expressed probe sets were identified using the limma package in Bioconductor project.

### Protein analysis through evolutionary relationships (PANTHER)

The PANTHER Classification System (supported by research grants from the National Human Genome Research Institute and the National Science Foundation, and maintained by the Thomas lab at the University of Southern California) was designed to classify proteins (and their genes) in order to facilitate high-throughput analysis. Details of the methods can be found in [27, 28]. PANTHER is part of the Gene Ontology Phylogenetic Annotation Project.

### Real time qRT-PCR

Total RNA was isolated from cultured lung epithelial cells isolated from patients (*n* = 11) and from sorted freshly isolated AT2- and AT1-like cells (*n* = 12) using the NucleoSpin RNA isolation kit with on-column DNase digestion (Macherey-Nagel, Düren, Germany). cDNA synthesis was performed using a High Capacity RNA-to-cDNA kit (Applied Biosystems, Thermo Fisher Scientific, Waltham, USA) following manufacturer’s protocols. For real-time qPCR experiments, master mixes with or without SYBR Green were used (Roche, Basel, Switzerland). Primer sequences are listed in Additional file 1: Table S2. PCR experiments were performed on a Light Cycler 480 Instrument (Roche, Basel, Switzerland). In the plots reverse dCt values versus GAPDH expression are presented; the following formula was used for calculation: dCt = Ct _target gene_-Ct_GAPDH._ Data was presented as relative quantity (RQ).

### 3D tissues and treatment with recombinant human Wnt proteins

Normal primary human small airway epithelial cells (SAEC) and normal human lung fibroblast (NHLF) were purchased from Lonza (Basel, Switzerland), isolated from anonymous donors of different ages and sexes. All cells were cultured at 37 °C and 5% CO_2_ in primary cell culture media. Both cell types were sub-cultured and mixed at 1:1 ratio then dispensed 3*10^5^ cells/well onto a low-attachment 96-well U-bottom plates (Corning, New York, USA) (Additional file 1: Figures S3 and S4). 3D aggregates were formed as described previously (Kovacs et al., 2014). Aggregates were treated with 0.1 μg/ml of recombinant human protein Wnt4, Wnt5a or Wnt7a, respectively for 48 h, then collected for total RNA isolation for TaqMan based PCR application (*n* = 3 biological repeats).

### Fluorescence staining of 3D aggregates

3D lung aggregates were embedded into TissueTek embedding media, frozen and 8 μm thick cryostat sections were cut and fixed in 4% para-formaldehyde (PFA) (Sigma-Aldrich, St. Louis, Missouri, USA). AQP5 was detected using an anti-AQ5 rabbit polyclonal IgG (sc-28,628) (Santa Cruz Biotechnology, Dallas, USA) (dilution 1∶100). ITGAV was detected using an anti-CD51 polyclonal goat antibody (PA5–47096), Thermo Fisher Scientific (Waltham, USA) (dilution 1:100). The secondary antibody was a goat anti-rabbit IgG antibody (Alexa Fluor® 568) (ab175471) (1:2000) (Abcam Plc, Cambridge, United Kingdom) and the anti-mouse antibody was an Alexa Fluor® 488 conjugated IgG (Thermo Fisher Scientific, Waltham, USA) (dilution 1:200). Nuclei were counterstained with Dapiprazole hydrochloride (DAPI)(ab142859) (1:1000) (Abcam Plc, Cambridge, United Kingdom). Images were acquired using Nikon Eclipse Ti-U microscope (Nikon GmbH CEE, Vienna, Austria) equipped with CCD camera (AndorZyla 5.5) then densitometry was performed using ImageJ.

### Artificial neural network (ANN) analysis

Evaluation of Wnt signaling pathway on AT2-to-AT1 TD was carried out using a feed forward artificial neural network (ANN) (Neurosolutions 6, NeuroDimension Inc.) software. Gene expression data were obtained with Affymetrix array using pooled cDNA samples of AT2 as controls and AT1 cell samples. Mean sensitivity was determined and set as to 1.0, all other sensitivity values are also shown accordingly in heat map format.

### Statistics

Statistical analysis was performed with SPSS version 20 software. Data are presented as mean ± standard deviation (STDEV), and statistical analysis was performed using Mann-Whitney non-parametric tests. *p* < 0.05 was considered as significant.

## Results

### Wnt signaling pathways are the most active during AT2-to-AT1 TD in vitro

**Fig. 1:**
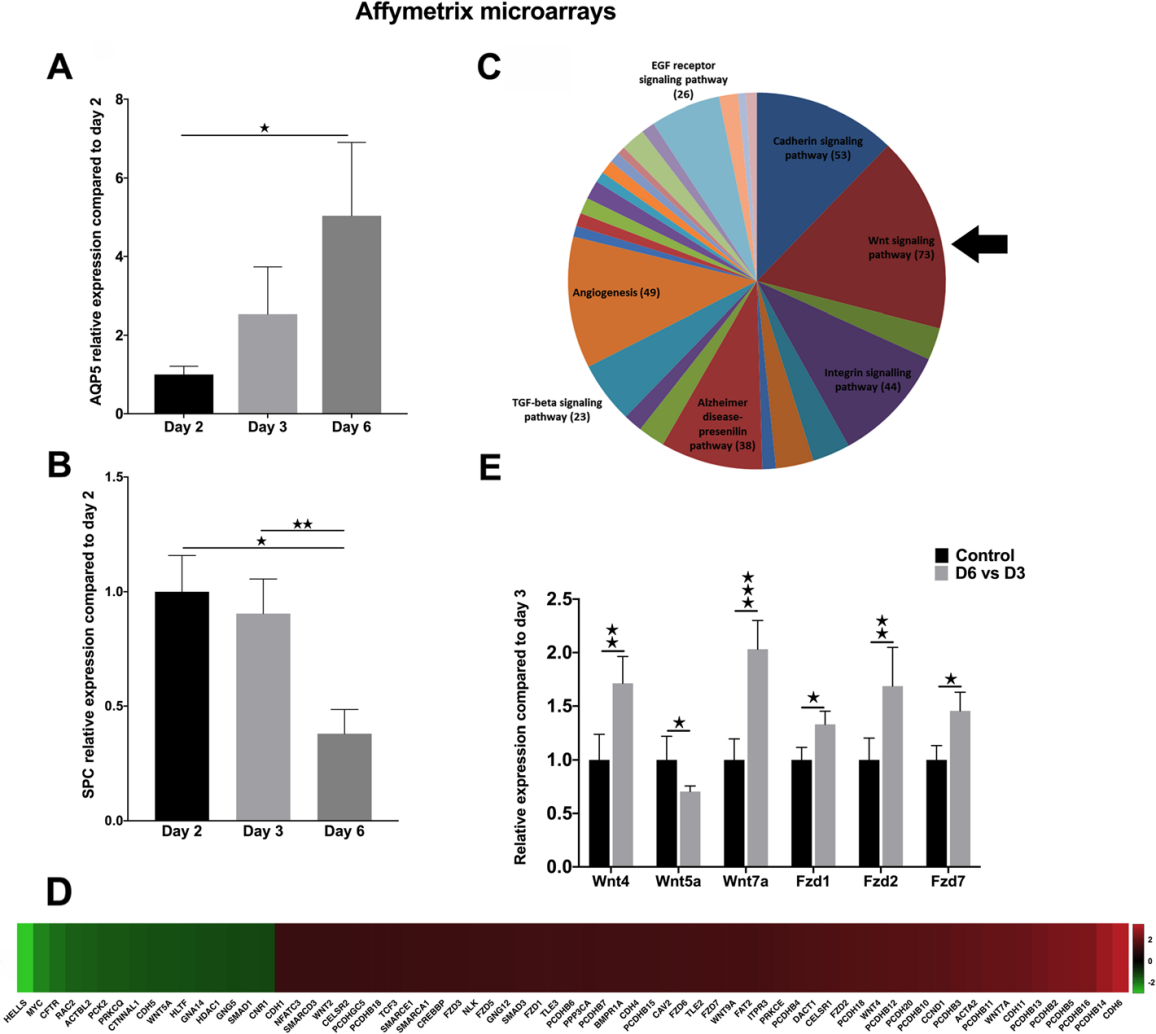
Gene expression changes during AT2-to-AT1 TD. Primary lung epithelial cells were isolated from lung tissue samples of lobectomy patients with normal lung function, then were cultured on collagen-coated plastic plates. mRNA was obtained on days 2, 3 and 6 of culture and real-time qPCR analysis was performed using Taqman® probes. GAPDH was used as a housekeeping gene. Affymetrix microarrays were also performed at each time points. **a** mRNA expression of AT1 marker AQP5 (*n* = 3/groups); **b** mRNA expression of AT2 marker SPC (*n* = 3/groups); **c** Summary of PANTHER analysis of Affymetrix microarray analysis. Numbers indicate the number of genes which expression is changed in day 6 of culture compared to day 3. **d**) Heat-map of Wnt signaling pathway-associated genes that are significantly (*p* < 0.01) changed during AT2-to-AT2 TD (*n* = 3). 73 genes associated with the Wnt pathway are changing significantly (*p* < 0.01) during the in vitro TD process. **e** Gene expression levels of Wnt ligands Wnt4, Wnt5a, Wnt7a and receptors Fzd1, Fzd2 and Fzd7 following in vitro TD using microarray (*n* = 3). Mann-Whitney test * *p* < 0.05, ** *p* < 0.01, *** *p* < 0.001

**Fig. 2:**
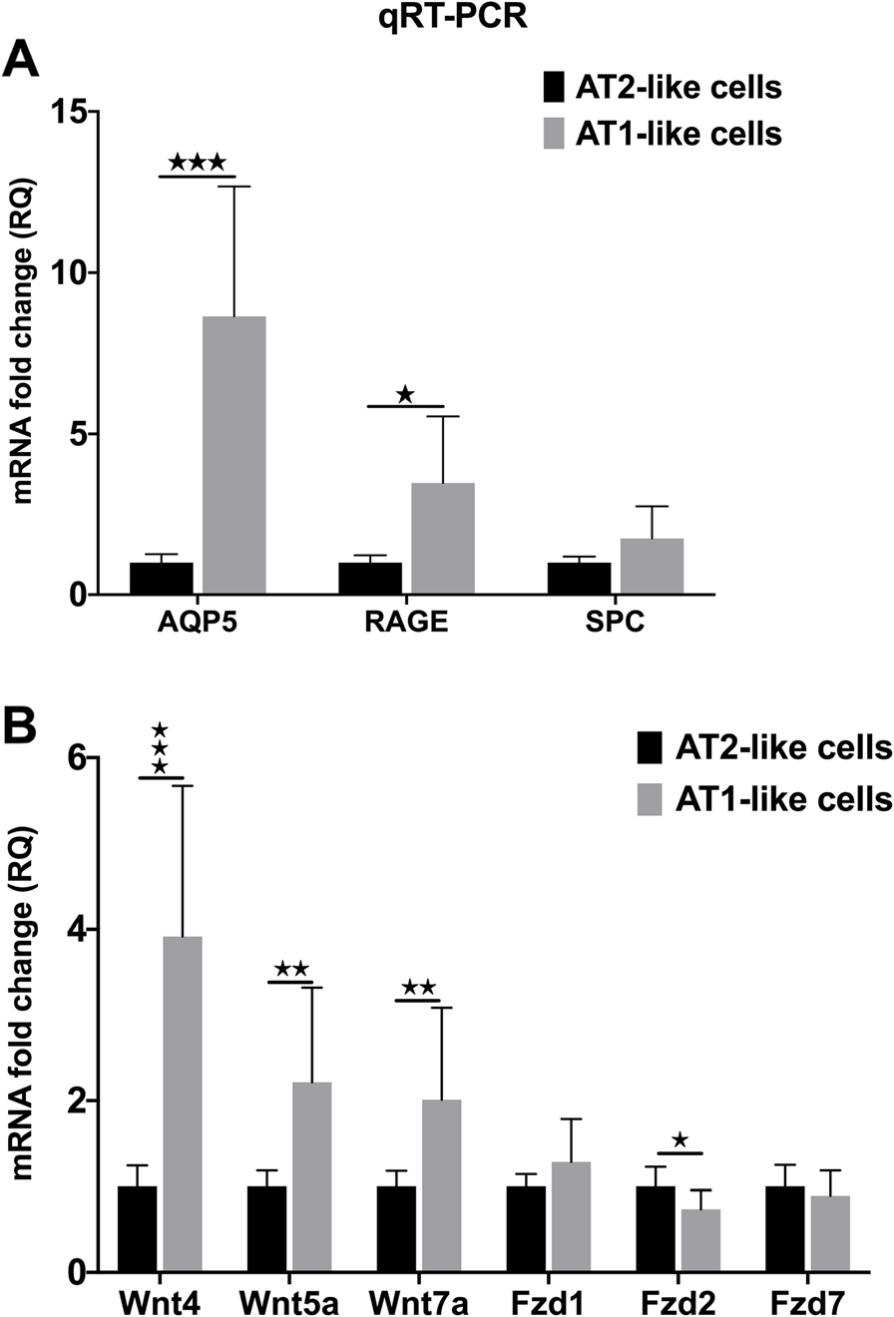
Gene expression analysis of freshly isolated and flow-sorted primary human lung epithelial cells. **a** Expression levels of AT1 and AT2 differentiation markers in freshly isolated human lung epithelial cells (*n* = 12) **b** Expression levels of Wnt ligands and Fzd receptors in freshly isolated lung epithelial cells (*n* = 12). Mann-Whitney test * *p* < 0.05, ** *p* < 0.01, *** *p* < 0.001. (See also Additional file 1: Figure S1 and S2)

Freshly isolated primary human AT2 cells were cultured in vitro for 2, 3 and 6 days, then mRNA was isolated and the generated cDNA was used in Affymetrix analysis. The AT1 marker AQP5 (Fig. 1a) and AT2 marker surfactant protein C (SPC) (Fig. 1b) were tested at each time points to confirm the initiation of the TD process. AQP5 levels increased dramatically while SPC levels decreased over time (Fig. 1a and b, respectively). To take an unbiased approach to pathway analysis, 1527 genes of an Affymetrix array were analyzed (donors *n* = 3). With the help of the Protein ANalysis THrough Evolutionary Relationships program (PANTHER) association studies revealed that amongst significant gene expression changes, 73 belonged to the Wnt pathway (Fig. 1c and d). mRNA levels of Wnt4, Wnt5a and Wnt7a ligands and Fzd1, Fzd2 and Fzd7 receptors changes were detected using the Affymetrix array (Fig. 1e) and confirmed by qRT-PCR analysis (Additional file 1: Figure S2). The in vitro detected gene expression changes were compared to mRNA levels of freshly isolated primary human AT1 and AT2 cells. Alveolar type identity of the sorted cell populations (Additional file 1: Figure S1) was confirmed by qRT-PCR analysis using differentiation markers SPC for AT2 (Fig. 2b), and AQP5/RAGE for AT1 (Fig. 2b). Although no significant differences were detected in SPC levels, AT1 markers (AQP5 and RAGE) were significantly higher in the sorted AT1 than in the AT2 population (Fig. 2b). While there was no remarkable difference in mRNA levels of Wnt receptors, all three Wnt ligands, Wnt4, Wnt5a and Wnt7a proved to be significantly higher in the freshly isolated primary AT1 cells than in AT2-s (Fig. 2c).

### Three dimensional (3D) aggregate cultures confirm a role of Wnt ligands in TD

**Fig. 3:**
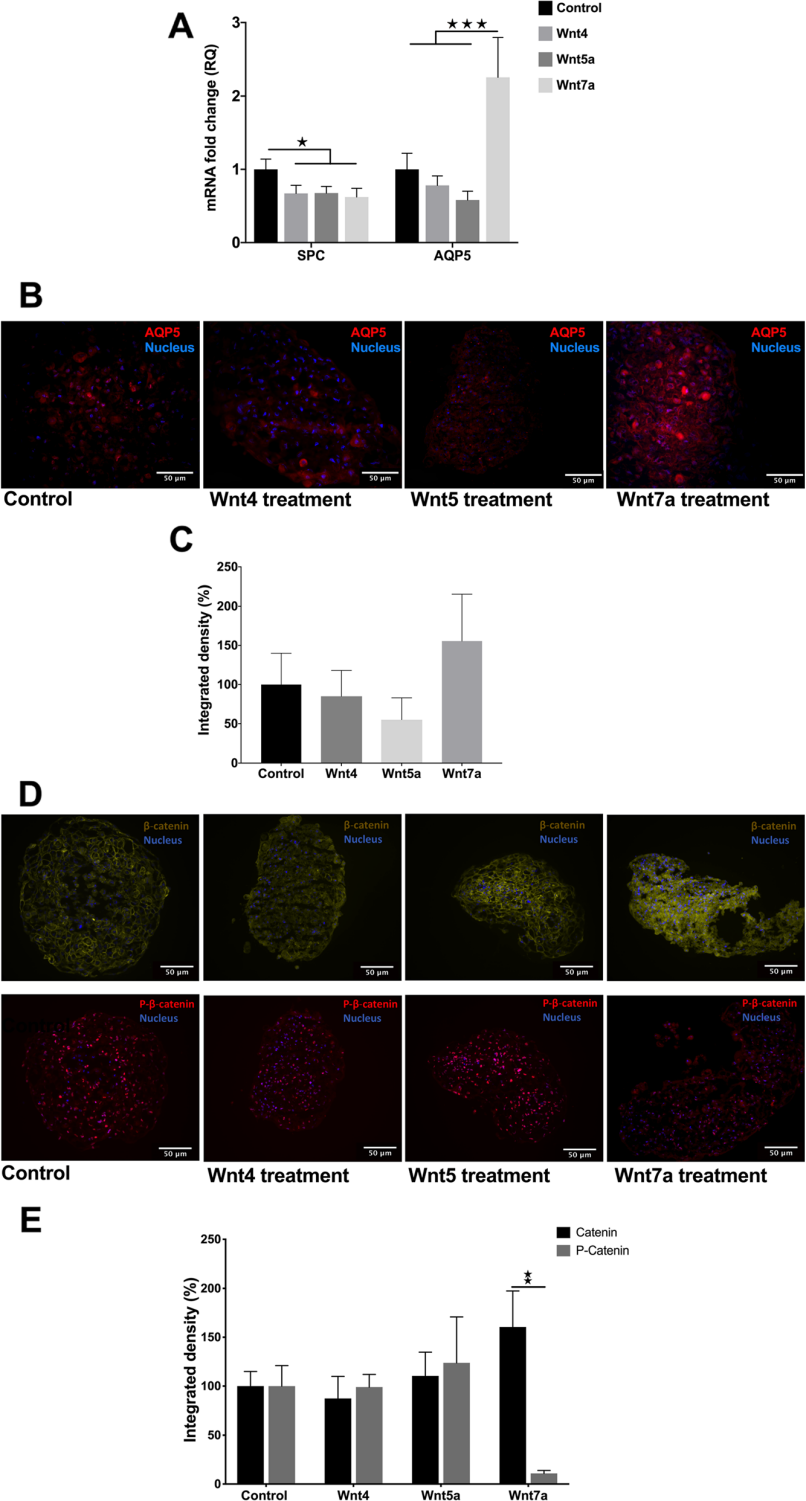
AT2 and AT1 marker expression in 3D aggregate cultures is regulated by Wnt ligands. **a** SPC is down- regulated by all Wnt4, Wnt5a and Wnt7a. Wnt7a upregulated the AT1 marker AQP5 (*n* = 3). **b** 3D lung aggregate tissue sections were stained with anti-AQP5 antibody (red), while nuclei were stained with DAPI (blue). Size bar is 50 μm. Representative of biological repeats, *n* = 3. **c** Densitometry of AQP5 staining (*n* = 3). Independent t-test, * *p* < 0.05. **d** 3D lung aggregate tissues were treated with Wnt7a ligands then sectioned and stained with anti-beta-catenin (pseudo-green) and anti-phospho-beta-catenin (red) antibody, respectively, while nuclei were stained DAPI (blue). Size bar is 50 μm. Representative of three independent biological repeats (*n* = 3). **e** Densitometry of beta-catenin and phospho-beta-catenin staining (*n* = 3, respectively). Mann-Whitney test * *p* < 0.05, ** *p* < 0.01, *** *p* < 0.001

To investigate the role of Wnt molecules in AT2-to-AT1 TD, in vitro 3D lung aggregate cultures [18] were generated using primary small airway epithelial cells (SAEC) co-aggregated with primary human lung fibroblasts (NHLF). In such culture systems SAECs undergo AT2-like differentiation and express SPC (Additional file 1: Figures S3 and S4) [18]. In the above system treatment with recombinant human (rh)Wnt4 or rhWnt5a downregulated SPC both at message and protein level (Additional file 1: Figure S3) [18]. Using the above aggregate culture system added rhWnt4, rhWnt5a and rhWnt7a triggered downregulation of SPC message levels, but only Wnt7a treatment increased the AT1 marker AQP5 both at mRNA (Fig. 3a) and protein levels (Fig. 3b, c). To investigate Wnt7a induced intracellular signaling activity, lung aggregate tissue sections were stained for beta-catenin and phospho-beta-catenin (Fig. 3d, e). Although beta-catenin levels increased upon Wnt7a exposure, activating beta-catenin phosphorylation (Ser675) levels decreased significantly, indicating the complexity of intracellular signal regulation that is involved in the TD process leading to AQP5 expression.

### ANN analysis of microarray data reveals Wnt pathway targets during AT2-to-AT1 TD

**Fig. 4:**
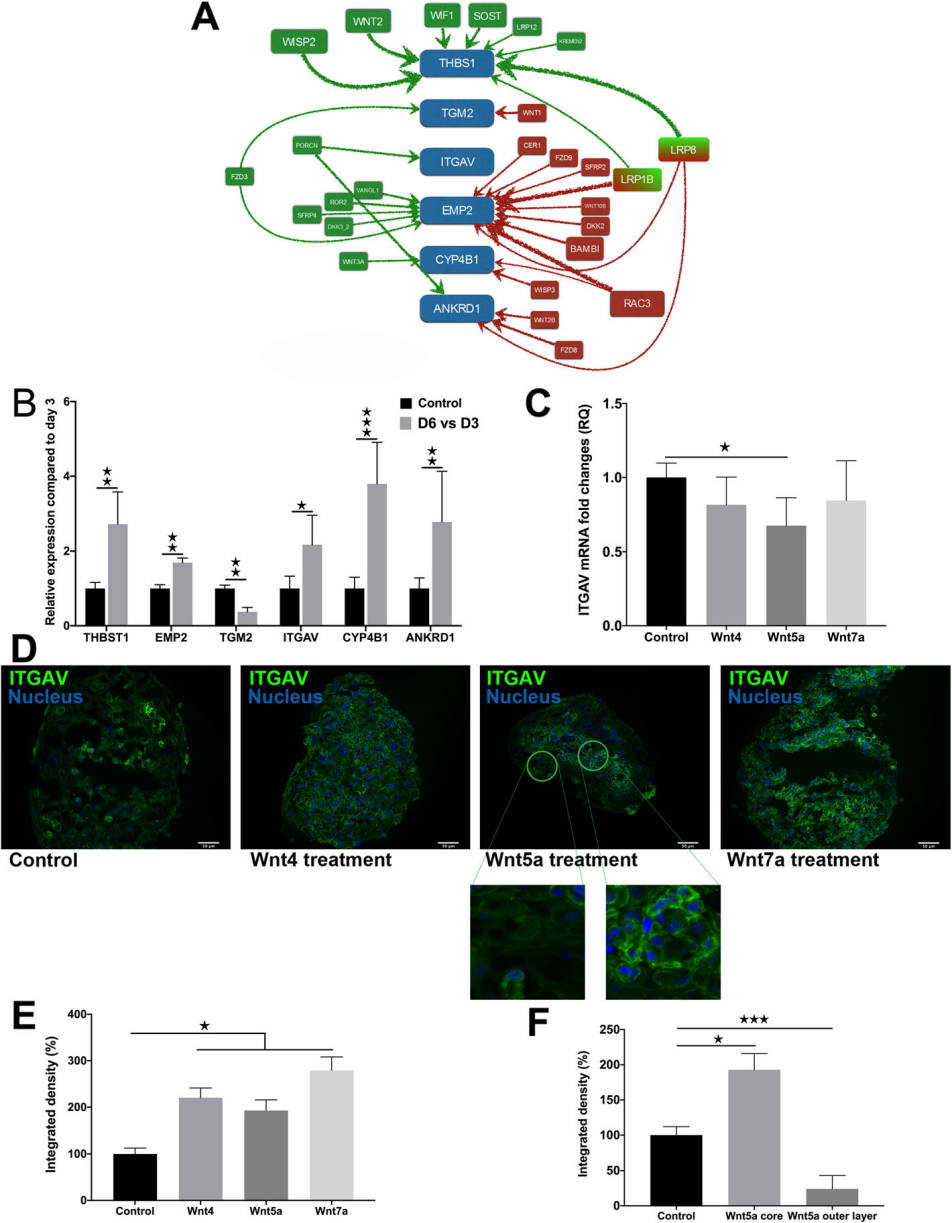
ANN analysis identifies target genes of Wnt signaling during AT2-to-AT1 TD. **a** Genes identified by ANN analysis depicting the strength of influence. **b** Gene expression of target genes measured by Affymetrix array. **c** Gene expression levels of Wnt pathway target genes measured in 3D lung re-aggregate cultures after Wnt ligand treatment (*n* = 3). Independent t-test, * *p* < 0.05. **d** 3D lung aggregate tissues were treated with Wnt4, Wnt5a and Wnt7a ligands then sectioned and stained with anti-ITGAV antibody (pseudo-green), while nuclei were stained DAPI (blue). Size bar is 50 μm. Representative of three independent biological repeats (*n* = 3). **e** Densitometry of ITGAV staining (*n* = 3). Independent t-test, * *p* < 0.05. **f** Densitometry of ITGAV staining (*n* = 3) of the core and the outer layer of the Wnt5a treated 3D lung tissue aggregate cultures (*n* = 3). Mann-Whitney test * *p* < 0.05, ** *p* < 0.01, *** *p* < 0.001

To discover molecular targets of the modified Wnt microenvironment, ANN analysis was performed that is a strong tool for predicting nonlinear associations (Fig. 4a). The analysis highlighted several molecules such as thrombospondin1 (THBS1), transglutaminase 2 (TGM2), integrin alpha V (ITGAV), epithelial membrane protein 2 (EMP2), cytochrome P450 family 4, subfamily B, polypeptide 1 (CYP4B1) and ankyrin repeat domain 1 (ANKRD1) as targets of the altered Wnt signaling pathway activity during the AT2-to-AT1 TD process (Fig. 4a). Based on the Affymetrix array analysis, mRNA levels of THBS1, EMP2, ITGAV, CYP4B1 and ANKRD1 were significantly increased, TGM2 significantly decreased during the TD process (Fig. 4b). Among these factors, THBS1 and EMP2 were affected the most (Fig. 4a). To link genes identified by the ANN analysis to specific Wnt ligands, 3D SAEC:NHLF aggregate co-cultures were treated with rhWnt ligands (rhWnt4, rhWnt5a and rhWnt7a) for 48 h (Fig. 4c), then qRT-PCR was performed on the generated cDNA, and tissue sections of the aggregates were stained for specific proteins. Only ITGAV mRNA was downregulated following rhWnt5a and not by rhWnt4 or rhWnt7a treatment indicating different targets of individual Wnt ligands. However, rhWnt5a treatment cell type specifically increased ITGAV protein levels (Fig. 4d, e, f) in the mesenchymal fibroblast core of the aggregate, while in the outer epithelial cell layer of the aggregate ITGAV protein levels significantly decreased (Fig. 4e and f).

## Discussion

In the present study, three Wnt ligands were identified to play important roles in the AT2-to-AT1 TD process, Wnt4, Wnt5a and Wnt7a. All three ligands were identified as down-regulators of SPC and Wnt7a as an inducer of the AT1 type differentiation marker AQP5. Previous studies support our discoveries. During the pulmonary aging process Wnt4 and Wnt5a were identified as inhibitors of lipid uptake and therefore surfactant production [18], while Wnt7a triggered differentiation and reduced proliferation of lung adenocarcinoma cell lines [29], respectively. The three Wnt ligands during AT2-to-AT1 TD are involved in a complex regulatory link with other genes identified by ANN. However, the only gene that was directly affected by an individual Wnt ligand, was ITGAV. ITGAV is strongly affected by PORCN (Porcupine) that is a membrane bound O-acyltransferase (MBOAT) involved in the acylation and secretion of Wnt proteins [30]. ITGAV in general plays an important role in the regulation of cancer growth, metastasis and tissue remodeling [31], but upregulation of ITGAV not just increases cellular adhesion but plays an inhibitory role in lipid transport that is essential for surfactant production [32]. As ITGAV is significantly increases during the TD process but decreases upon Wnt5a ligand treatment, it was assumed that elevated levels of ITGAV aids AT1 differentiation via blocking surfactant production. Additional analysis of ITGAV protein levels have, however, demonstrated that Wnt5a can cell type specifically modify ITGAV expression. While in the mesenchymal fibroblasts ITGAV levels increased, in the epithelial cell layers ITGAV levels significantly decreased corresponding to decreased mRNA levels in the aggregate cultures following rhWnt5a treatment. Such results support previous findings that Wnt5a triggers ITGAV expression in the mesenchyme [33] and also that SPC production is associated with fibroblast differentiation [18]. Consequently, we can hypothesize that ITGAV and not directly Wnt ligands are responsible for regulation of SPC levels.

The other genes identified by ANN analysis are more difficult to explain as in follow-up experiments neither Wnt4, Wnt5a or Wnt7a affected individually the expression of THBS1, TGM2 or EMP2. The most strongly affected by the modified Wnt microenvironment is the up-regulated THBS1, that is a secreted glycoprotein involved in wound healing, angiogenesis and inflammatory response [34] as well as in inhibition of tumor growth [35]. Upregulation of THBS1 during a physiological regeneration process could be a built-in molecular protection mechanism against carcinogenesis. The significantly downregulated TGM2 gene encodes an ubiquitously expressed enzyme capable of catalyzing protein cross-links and regulate extracellular matrix integrity [36]. Down-regulation of TGM2, however, fits into the envisaged TD process, as loosening the extracellular matrix is probably needed to facilitate AT2 spreading over the basal membrane. Increased expression of EMP2 has been linked to cancer progression by controlling cell membrane composition [37] and blood vessel growth [38]. So, elevated EMP2 expression during the physiological AT2-to-AT1 TD potentially facilitates capillary blood vessel formation. Additionally, ANN revealed that EMP2 is most sensitive to inhibitors of the canonical and the PCP Wnt pathways like VANGL1, DKK2, SFRP4, receptors like ROR2 and the Wnt2 associated receptor, Fzd3. Simultaneously, EMP2 is unaffected by a number of genes with similar inhibitory characteristics (CER1, SFRP2, Fzd9 and BAMBI) indicating the existence of a so far unidentified regulatory network of alveolar regeneration. Finally, upregulation of CYP4B1 is also a characteristic marker of cellular –mainly bronchial- differentiation of the lung [39]. Upregulation of CYP4B1 during AT2-to-AT1 TD is affected by a specific ligand Wnt3a, that plays an important role in in lung cancer [40].

## Conclusions

Investigation of gene expression during AT2-to-AT1 TD not only identified Wnt ligands that can accelerate AT1 type differentiation. We have also identified Wnt pathway associated genes that are affected by the cumulative changes in the Wnt microenvironment. The balance of the microenvironment, however, is crucial as most of the target genes are important regulators of carcinogenesis or cancer progression. In the light of our research data it is not surprising that in recent years Wnt signaling has become a target of investigation for both cancer [41] and regenerative therapies [42].

## Supplementary information file

Additional file 1: **Figure S1.** Flow-sorting of freshly isolated primary lung epithelial cells using a triple-labeling technique. Related to Fig. 2. **Figure S2.** Gene expression analysis of freshly isolated and flow-sorted primary human lung epithelial cells. **Figure S3.** In untreated SAEC-NHLF (1:1) aggregate co-cultures SEAC-s produce SPC. **Figure S4.** Localization of primary SAEC and NHLF in aggregate tissue cultures. **Table S1.** Patient characteristics. **Table S2.** Primer sequences. (DOCX 16693 kb)

## Data Availability

All large datasets generated and analyzed during the current study are available from the corresponding author on reasonable request.

## Abbreviations

ANKRD1: Ankyrin repeat domain 1
ANN: Artificial neural network
AQP5: Aquaporin 5
AT1: Alveolar Type 1 Cell
AT2: Alveolar Type 2 Cell
BAMBI: BMP and activin membrane-bound inhibitor
BMP: Bone morphogenic protein
CER1: Cerberus1
CYP4B1: Cytochrome P450 family 4, subfamily B, polypeptide 1
DAPI: Dapiprazole hydrochloride
DKK2: Dickkopf-2
EMP2: Epithelial membrane protein 2
EMT: Epithelial mesenchymal transition
EPCAM1: Epithelial Cell Adhesion Molecule
FACS: Fluorescent activated cell sorting
FGF: Fibroblast growth factor
Fzd: Frizzled
ITGAV: Integrin alpha V
KRE: Kremen
MBOAT: Membrane bound O-acyltransferase
NHLF: Normal Human Lung Fibroblasts
PANTHER: Protein ANalysis THrough Evolutionary Relationships program
PORCN: Porcupine
RAGE: Receptor for Advanced Glycation End-Products
ROR2: Receptor Tyrosine Kinase Like Orphan Receptor 2
SAEC: Small airway epithelial cells
SFRP2,4: Soluble frizzled receptor peptide
SPA: Surfactant protein A
SPC: Surfactant protein C
TD: Trans-differentiation
TGM2: Transglutaminase 2
THBS1: Thrombospondin1
VANGL1: VANGL planar cell polarity protein 1
WISP: WNT1 Inducible Signaling Pathway Protein
