# Supplementary material for: Wnt signaling regulates trans-differentiation of stem cell like type 2 alveolar epithelial cells to type 1 epithelial cells
Source: Respir Res. 2019 Sep 6;20:204. doi: 10.1186/s12931-019-1176-x (PMC6731587; doi:10.1186/s12931-019-1176-x)
Supplement: Supplementary file 1 — Figure S1. Flow-sorting of freshly isolated primary lung epithelial cells using a triple-labeling technique. Related to Fig. 2. Figure S2. Gene expression analysis of freshly isolated and flow-sorted primary human lung epithelial cells. Figure S3. In untreated SAEC-NHLF (1:1) aggregate co-cultures SEAC-s produce SPC. Figure S4. Localization of primary SAEC and NHLF in aggregate tissue cultures. Table S1. Patient characteristics. Table S2. Primer sequences. (DOCX 16693 kb) [file 12931_2019_1176_MOESM1_ESM.docx]

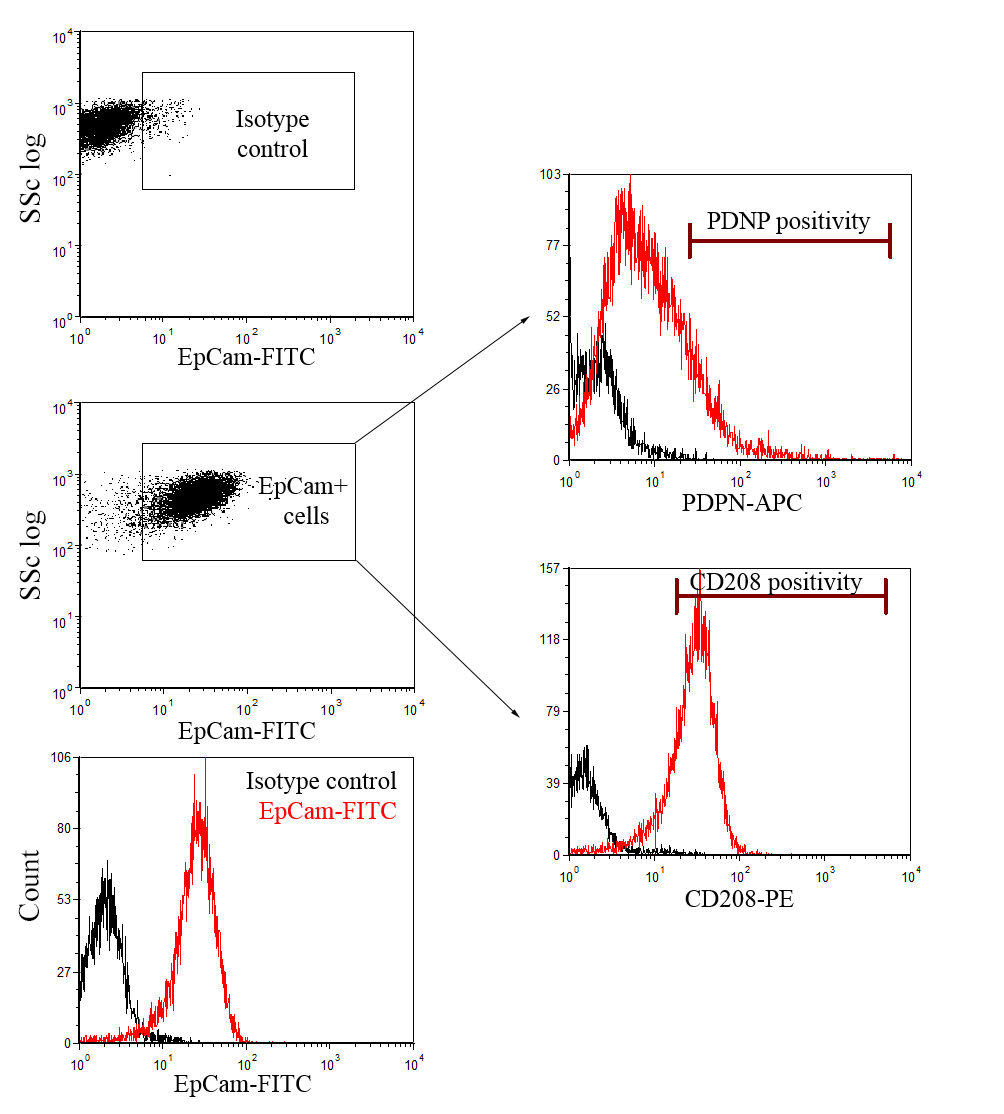


**Figure S1.: Flow-sorting of freshly isolated primary lung epithelial cells using a triple-labeling technique. Related to Figure 2.**

AT2- and AT1-like cells were isolated using a triple-labelling technique. Lungs of lobectomy patients with normal lung function were labelled with antibodies specific to EpCAM1+, CD208+ and Podoplanin+. EpCAM1+, CD208+ double positive cells are AT2-like cells and EpCAM1+ Podoplanin+ cells are AT1-like cells. The average cell yield of AT2-like cells were 3.13x higher than that of AT1-like cells (n=12).


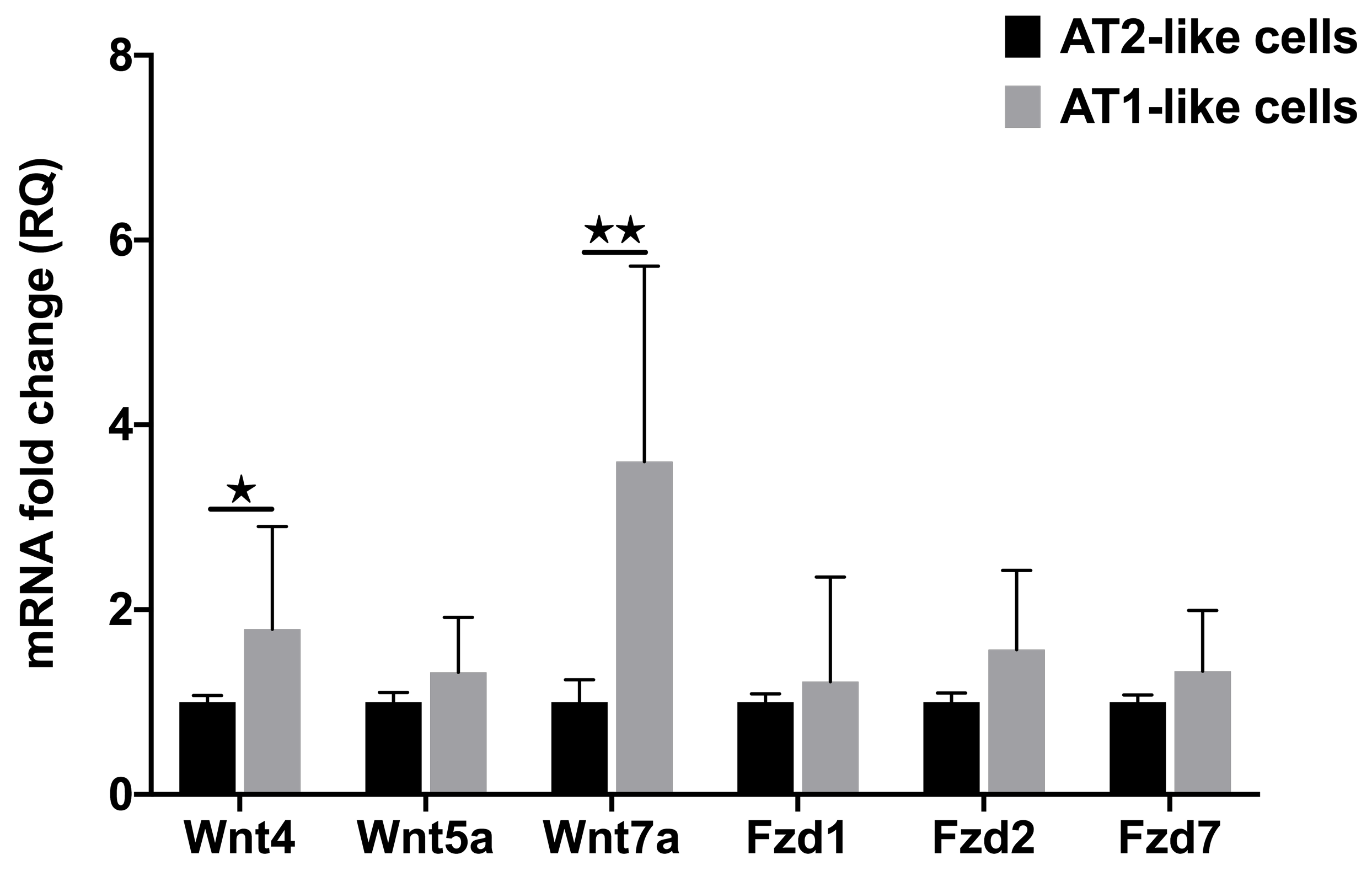


**Figure S2.: Gene expression analysis** **of freshly isolated and flow-sorted primary human lung epithelial cells.** qPCR confirmation of selective Wnt molecules on in vitro AT2-to-AT1 transdifferentiated cells (n=11). * p<0.05, ** p<0.01.


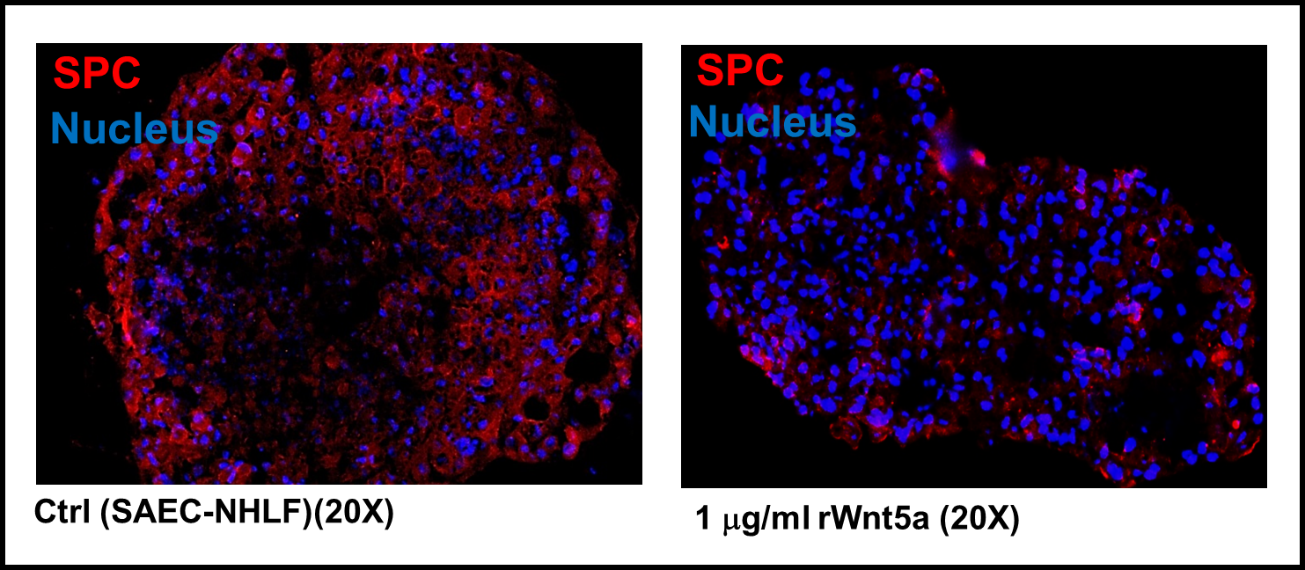


**Figure S3.: In untreated SAEC-NHLF (1:1) aggregate co-cultures SEAC-s produce SPC.** Treatment with 1 µg/ml Wnt5a reduces SPC production within 48 h of culture.


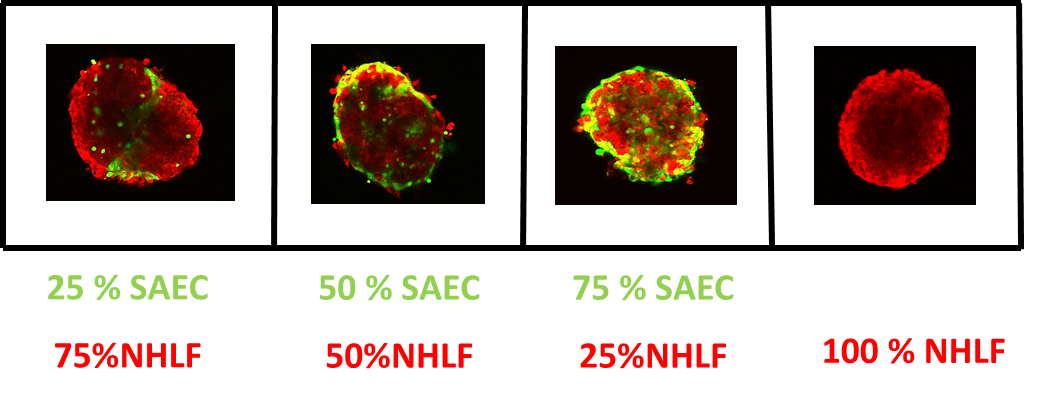


**Figure S4.: Localization of primary SAEC and NHLF in aggregate tissue cultures.** Cells were stained with the vital stains of Dil and CFSE (using 1:1000 dilutions of physiological fluorescent dyes of Dil (1mg/ml stock in DMSO) and CFSE (1mg/ml stock in DMSO) in PBS (phosphate buffer saline pH 7.2)).

**Tables**

| Male/Female ration | 60/40 |
| --- | --- |
| Number of patients | 12 |
| Ex-smokers | 4 |
| Non-smokers | 8 |
| Excluded patients | COPD |
| Mean age (yrs) | 67 |

**Table S1. Patient characteristics**

| **Primers** | **Forward** | **Reverse** |
| --- | --- | --- |
| Advanced glycosylation end-product specific receptor (RAGE), Homo sapiens | CCGAGTCCGTGTCTACCAGATT | CACATGTCCCCACCTTATTGG |
| Ankyrin repeat domain 1 (ANKRD1), Homo sapiens | AGAACTGTGCTGGGAAGACG | GCCATGCCTTCAAAATGCCA |
| Aquaporin 5 (AQ5), Homo sapiens | TCATGAATCGGTTCAGCCCC | GTCCTCGTCAGGCTCATACG |
| Cytochrome P450 family 4 subfamily B member 1 (CYP4B1), Homo sapiens | CCTCTCCCTGAGCTTCTCCT | CAGCAGGTGGATGAGCTTGA |
| Epithelial membrane protein 2 (EMP2), Homo sapiens | CAGCCCTCACTTGGTGCTTA | TGCTCTGGGCAATCTGGTTT |
| Frizzled class receptor 1 (Fzd1), Homo sapiens | TAATTGACGGGGAGATAGCG | GACCTGCACACATTTTCCCT |
| Frizzled class receptor 2 (Fzd2), Homo sapiens | ATTTTTCTGTCGGGCTGCTA | TCATGAAGAGGATGGTGCAG |
| Frizzled class receptor 7 (Fzd7), Homo sapiens | CGACGCTCTTTACCGTTCTC | CCATGCCGAAGAAGTAGAGC |
| Integrin subunit alpha V (ITGAV), transcript variant 1,3, Homo sapiens | GGGAAGCAAAGGACCGTCTG | ATGGTACAATGGGGCACAGG |
| Surfactant protein A (SFTPA), Homo sapiens | TGTCCTCAATTTCATTGCCA | CGGTCACATCAGATCAGTGG |
| Surfactant protein C (SFTPC), Homo sapiens | AAAAGTCCACAACTTCCAGGC | GATGTAGTAGAGCGGCACCT |
| Thrombospondin 1 (THBS1), Homo sapiens | CCATGCTTATTTGTTCTCTACTGGC | GGCCTGAGCAACTCAGTCTT |
| Transglutaminase 2 (TGM2), Homo sapiens | ATAAGTTAGCGCCGCTCTCC | TAGTTGTTGTCCCAGCGTCC |
| Wingless-type MMTV integration site family, member 4 (Wnt4), Homo sapiens | CCTTCGTGTACGCCATCTCT | GCCTCATTGTTGTGGAGGTT |
| Wingless-type MMTV integration site family, member 5a (Wnt5a), Homo sapiens | TGGCTTTGGCCATATTTTTC | CCGATGTACTGCATGTGGTC |
| Wingless-type MMTV integration site family, member 7a (Wnt7a), Homo sapiens | AGTACAACGAGGCCGTTCAC | ACAGCACATGAGGTCACAGC |

**Table S2.: Primer sequences**
